# An exploration of current and future vector-borne disease threats and opportunities for change

**DOI:** 10.3389/fpubh.2025.1585412

**Published:** 2025-05-15

**Authors:** Alexandra Hiscox, Robert T. Jones, Jessica Dennehy, Will Dyall, Laura Paris, Freya I. Spencer, Fiona Keating, Frederik Seelig, Abhinandan Narendran, Avijit Das, James G. Logan

**Affiliations:** ^1^Arctech Innovation, Dagenham, United Kingdom; ^2^Department of Disease Control, London School of Hygiene and Tropical Medicine, London, United Kingdom; ^3^Center for Immunology of Viral Infections, Department of Biomedicine, Aarhus University, Aarhus, Denmark; ^4^Reckitt Benckiser (India) Pvt. Ltd., Gurgaon, Haryana, India

**Keywords:** vector-borne disease, climate, Brazil, India, surveillance

## Abstract

Vector-borne diseases, including dengue, threaten the health and livelihoods of over 80% of the world’s population, particularly in tropical and subtropical regions. Environmental, ecological, climatic, and socio-economic factors are expected to drive increased transmission, emphasizing the need to identify key threats and prioritize strategies for control. We examined drivers, challenges and potential solutions with global experts, using Brazil and India as case studies. Both countries face rapid population growth, unplanned urbanization and increased exposure to animal reservoirs alongside unique surveillance and control challenges. We advocate for improvements in surveillance systems and capacity, investment in sustainable vector control tools, leveraging of artificial intelligence for outbreak prediction, and fostering public-private partnerships to develop innovative interventions. A multifaceted approach, combining community-led initiatives with advanced technologies, is essential to reducing the burden of vector-borne diseases and preventing future epidemics.

## Introduction

Vector-borne diseases (VBDs) account for 17% of all infectious diseases, and lead to 700,000 deaths every year (1). A review of all emerging disease events at the start of the twenty-first century revealed that almost 30% were vector-borne, and the emergence of novel pathogens has correlated significantly with environmental, ecological, climatic and socio-economic factors (2, 3). Now is a critical time to identify which diseases pose the greatest future health threats and focus on the most promising strategies to limit their impact now and in the future. We explored these issues in interviews with global experts in VBDs to understand the most pressing concerns. The thoughts of these stakeholders are presented using the countries of Brazil and India as case studies.

## Burden of vector-borne diseases in Brazil and India

Brazil has the highest burden of VBDs across all Latin America and the Caribbean and has experienced a significant number of outbreaks this century. Most importantly, there has been an increase in the number of dengue epidemics and the geographical range of this disease (4, 5). In 2023 there were over 1.6 million probable cases of dengue across Brazil and over 1,000 deaths, and in 2024 those figures stood at over 6.5 million probable cases and more than 6,000 deaths, underscoring the dramatically increased risk of arbovirus infections in a short span of time (6). There were also over 260,000 probable cases of chikungunya in 2024, and over 13,800 confirmed cases of Oropouche virus infection (6).

At the start of this current rapid increase in dengue, Dr. Aline Campos, Chief of Environmental Health Surveillance Division and the State Department of Health, Brazil, told us of her state, Rio Grande do Sul: *“The VBD of most concern for public health is dengue, absolutely. Dengue is the major problem here. We also have chikungunya. We also have Zika. But all the deaths this year (2022) were related to dengue.”* This view was supported by Dr. Marcio Pavan, Associate Professor, Laboratório de Mosquitos Transmissores de Hematozoários, Fundação Oswaldo Cruz: “*The arboviruses are the most worrisome VBDs because they are in areas of high human population density and present important sequelae. For example, Zika and microcephaly in newborns, or Guillain-Barré syndrome and chikungunya, or dengue and hemorrhagic conditions. Many infections lead to death*.” *Aedes*-borne arboviruses – dengue, chikungunya, yellow fever and Zika – are considered the diseases most likely to cause epidemics in the future due to the anthropophilic nature of their vectors, which are widespread in tropical and sub-tropical regions (7). Brazil also has the highest number of confirmed cases of Mayaro virus infection in Latin America and the Caribbean, and other vector-borne pathogens of concern include Oropouche virus, which has been responsible for over half a million human cases in Brazil to date (8, 9).

Major VBDs circulating in India include malaria, dengue, chikungunya, Japanese encephalitis, lymphatic filariasis, visceral leishmaniasis, and plague (10–12). In 2024, the National Centre for Vector Borne Diseases Control reported 236 deaths and over 233,000 cases of dengue, as well as more than 231,000 suspected and 17,800 confirmed cases of chikungunya (13, 14). India harbors nearly 40% of all global lymphatic filariasis infections, with 619,000 lymphedema and 126,000 hydrocele cases reported as of 2023 (15). It also accounts for approximately 18% of worldwide cases of visceral leishmaniasis, known locally as kala-azar (16, 17), reporting 438 cases and 2 deaths in 2024 (18). Whilst progress has been made toward elimination, Professor Mary Cameron, Professor of Medical Entomology, the London School of Hygiene & Tropical Medicine (LSHTM), warned that the country must remain vigilant: “*A significant challenge is that once elimination targets have been reached, people take the foot off the brake. So, there remains the possibility that a disease like visceral leishmaniasis can cause a huge outbreak if you do not have good surveillance systems in place.”*

## Climate change

Climate change and urbanization were most frequently mentioned by our experts as main contributors to the emergence and spread of VBDs. In Brazil as well as other areas of the tropics, increases in temperature, rainfall and duration of wet seasons can enhance opportunities for pathogens and vectors to reproduce at higher rates and transmit pathogens over broader geographical ranges for more months of the year. Extended wet seasons may increase opportunities for spatial and temporal overlap and interaction between vectors, pathogens and novel hosts, and this could lead to the emergence of a disease or the spillover of a pathogen to a new vector or host population (19). Simulation models project that approximately half of the global population may be exposed to *Aedes aegypti* by 2050, and that 60% of the world’s population will be at risk of dengue by 2080 (20, 21).

The number of months suitable for dengue transmission in India has increased over the last half-century, with *Aedes* population growth being enabled by more favorable climatic conditions (22). Models predict that coastal regions may experience year-round transmission of vector-borne diseases in the future, though the highest transmission potential will continue to occur during the monsoon season (23). Along the coast, rising sea levels have caused an expansion of brackish and saline water bodies. *Culex tritaeniorhynchus* and *Cx. gelidus*, which are competent vectors of Japanese encephalitis and West Nile virus, are becoming increasingly tolerant of saline conditions and can now breed in salty water bodies, which has huge implications for VBD control in India’s coastal regions (24). Public health authorities must recognize and acknowledge the impact that such adaptations can have on disease transmission, and institute appropriate surveillance and control measures.

## Environmental, ecological and behavioral drivers

Other environmental and ecological factors driving the spread of VBDs were also raised, including changes in land use and increased contact between people and nature. Models exploring the spatiotemporal variation of yellow fever both inter-annually and seasonally suggest that heterogeneity in vegetation (a proxy for habitat fragmentation) and land cover could explain both the expansion of the yellow fever transmission zone as well as the highly seasonal nature of yellow fever in Brazil (25). Globally, agricultural drivers have been associated with more than 25% of emerging infectious diseases and more than 50% of emerging zoonotic diseases in humans (26). Some agricultural practices such as the expansion of habitable cropland, and other microclimatic land use changes can lead to the creation of larval habitats for vectors or increase contact between sylvatic vectors, humans and livestock (27). Dr. Emma Maynard, Research Manager, Infections and Climate, Wellcome Trust, explained: “*When the natural environment is altered to facilitate a different land use, such as mining, agriculture or dams, humans are brought into closer contact with wildlife, vectors and potential infectious disease reservoirs. The local climate and environmental conditions change, which can influence vector abundance and behaviour facilitating increased disease transmission.”*

Changes in land use occurring with the expansion of urban environments have been associated with the emergence and spread of those VBDs whose vectors thrive in and around human dwellings (28). In Brazil, rapid urban population growth has included informal settlements that lack the basic infrastructure and services that are important for controlling *Aedes* populations, such as drainage systems and waste management (29).

**Figure 1:**
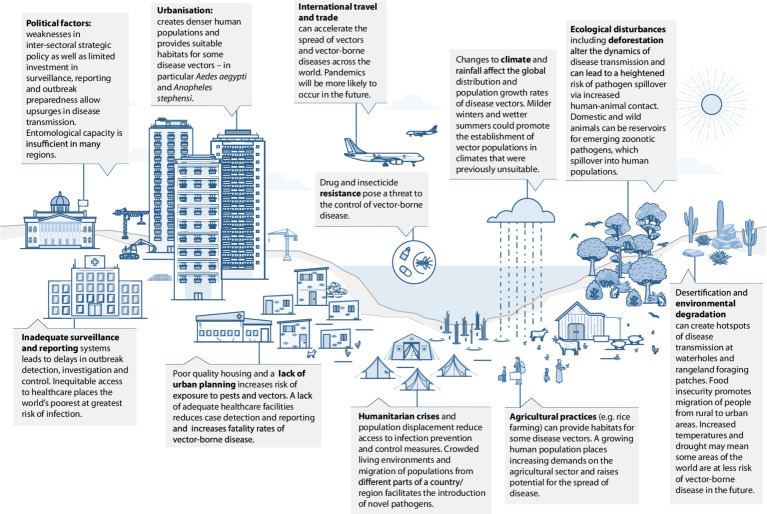
Factors contributing to the emergence and spread of vector-borne diseases.

In India, human migration has been a major factor contributing to malaria transmission, especially in cities where there have been influxes of migrant workers from rural parts of the country, driven in-part by climate change and associated challenges faced by agricultural communities (30). Mobile populations may be undocumented and have limited access to health services (31), so pose a challenge to disease control and elimination. India is home to some of the largest religious and cultural mass gatherings in the world and disease outbreaks have occurred during several of these events, contributing to disease transmission in other parts of the country once visitors return home (32). It is likely that these large-scale gatherings provide platforms for exchange of genomic material and thereby evolution of pathogens, including viruses. Dr. Arun Sivan, Consultant Entomologist (Technical), National Vector Borne Disease Control Programme, Odisha, identified this among other concerns in India: “*The main contributors to the emergence and spread of new and existing VBDs are rapid and unplanned urbanization, and increased migration of people for occupation, tourism, and pilgrimage*” (Figure 1).

## Surveillance and control

India’s Directorate of National Vector Borne Disease Control Programme (NVBDCP) is responsible for formulating technical guidance and policy-making to guide states in the implementation of disease prevention and control programs. Each state in India has a VBD control unit responsible for leading prevention, surveillance and control in their sub-districts (33), but the VBD surveillance system in India needs enhancement, especially in urban areas, and could benefit from integrated data from both private and public healthcare services (34). There is also an urgent need for human resources, including trained medical entomologists. A demand–supply analysis estimated the need for at least a thousand specialists in medical entomology in India, while annual output is typically below one hundred (35). Dr. Arun Sivan: “*Shortcomings in the ability to survey and control VBDs include a lack of trained manpower, especially entomologists*.”

There are opportunities to support surveillance and control through community engagement and involvement in control activities. Dr. Melinda Rostal, Principal Scientist, Vector-Borne Diseases, EcoHealth Alliance told us: “*It is critical to think about integrating social science into the development of interventions involving personal protection. This includes taking the time to understand how the community understands VBDs and the best way to present the intervention to said community and maximize uptake of the intervention.”* Dr. Helen Jamet, Deputy Director, Malaria, Bill & Melinda Gates Foundation, agreed: *“Most personal protection tools unfortunately rely on behavioural change and adherence, which can limit their efficacy even if the individual tool works very well.”*

Community-led environmental and larval site management methods have proven to be effective methods of vector control (34). As Dr. Raman Velayudhan, of the Global Neglected Tropical Diseases Programme, World Health Organization, identified: ultimately, “*a combination of tools will be needed to control VBD outbreaks in the future including multiplex diagnostics, better case management, innovative sustainable vector control tools targeting both immatures and adults, effective vaccines, therapeutics, integrated surveillance and above all community support.”* Dr. Oliver Brady, Associate Professor, LSHTM told us that “*new tools need to be sustainable (ideally with minimal ongoing effort), synergistic with existing interventions and have strong evidence (ideally cluster randomized trial(s)) of reducing cases of disease, not just entomological outcome(s)*.”

## Discussion

Dengue and other *Aedes*-borne diseases will continue to be a significant health burden in tropical and subtropical regions of the world, and climate change, increasing urbanization, population movement and land-use change will drive the emergence and spread of VBDs. Prioritizing funding for national surveillance and data reporting systems could be an effective way to fill gaps in knowledge, enabling more accurate prediction of disease occurrence and better allocation of resources to prevent and control outbreaks. Countries should strengthen community engagement activities and education initiatives, ensuring those most affected or at greatest risk of disease are supported in adopting effective prevention measures. Governments should be open to the use of social media, citizen science and non-standard data to inform detection and early warning systems, and to public-private partnerships that can innovate and accelerate the creation of more sustainable, more efficacious tools for control, with a focus on new active ingredients and more environmentally conscious approaches to manufacturing. Industry can play a critical role in scale-up and integration of vector control tools through leveraging supply chain management and offering expertise in logistics, cost-efficiencies, and long-term planning capabilities (36). Advances in artificial intelligence and machine learning should also be embraced. These could support improvements in models and enable public health policy makers to generate outbreak preparedness plans, including effective allocation of limited health resources to tackle diseases in hotspots of transmission.

Human populations will only be free of the threat of VBDs if they can be eliminated. This is an enduring challenge, particularly in the face of resistance to chemical tools. Whilst some countries have achieved eradication of malaria or other VBDs in their territories, their citizens now, or will soon, face the risk of other infections. It is vital that we accelerate our understanding of the risks, that we push forward new ideas at pace, and take ownership of our diverse roles to create robust methods to predict, prevent and manage these diseases now and for the future.

## Data Availability

The raw data supporting the conclusions of this article will be made available by the authors, without undue reservation.
